# Hyperflexing the horse’s neck: a systematic review and meta-analysis

**DOI:** 10.1038/s41598-024-72766-5

**Published:** 2024-10-02

**Authors:** Uta König von Borstel, Kathrin Kienapfel, Andrew McLean, Cristina Wilkins, Paul McGreevy

**Affiliations:** 1 Department of Animal Breeding and Genetics, Section Animal Husbandry, Behaviour and Welfare, University of Giessen, Leihgesterner Weg 52, 35392 Giessen, Germany; 2 Group Equids, Swiss national stud farm, Les Longs Pres, Agroscope, Avenches, 1580 Switzerland; 3 Equitation Science International, 3 Wonderland Avenue, Tuerong, VIC 3915 Australia; 4 School of Environmental and Rural Science, University of New England, Armidale, NSW 2353 Australia; 5 Sydney School of Veterinary Science, University of Sydney, Armidale, NSW 2006 Australia

**Keywords:** Animal behaviour, Animal physiology, Biomechanics, Musculoskeletal system

## Abstract

The article reports a meta-analysis of 58 peer-reviewed studies investigating on dorsoventral hyperflexion of the neck in horses, a practice under substantial public and scientific scrutiny for the past two decades. The following databases were last searched on 28.05.2023: CAB, Google Scholar, Web of Science, NAL/Agricola, PubMed and ScienceDirect. After evaluating the conclusions of each study, we performed statistical analyses to establish a consensus on welfare and performance (performance marks, kinematics and musculoskeletal) outcomes in horses performing with a hyperflexed head and neck posture (HNP). The analysis revealed that a significant majority of the articles (75% of *n* = 36; Z = 3.00; P>|Z|=0.0027) expressed concerns about the welfare of horses working in this posture. Parameters such as dressage training level, prior experience, duration, and method of achieving the hyperflexed posture did not influence welfare concern conclusions significantly (*P* > 0.1). Therefore, it appears that the practice impairs welfare regardless of how it is imposed. A concurrent assessment of the weight of evidence for performance benefits showed inconclusive results: approximately one-quarter of the studies showed benefits and one-quarter detrimental effects, while the largest proportion (44%) showed no significant effect on performance. On balance, it appears that the costs associated with hyperflexed HNPs exceed potential benefits.

**Supplementary Information:**

The online version contains supplementary material available at 10.1038/s41598-024-72766-5.

## Introduction

**Fig. 1:**
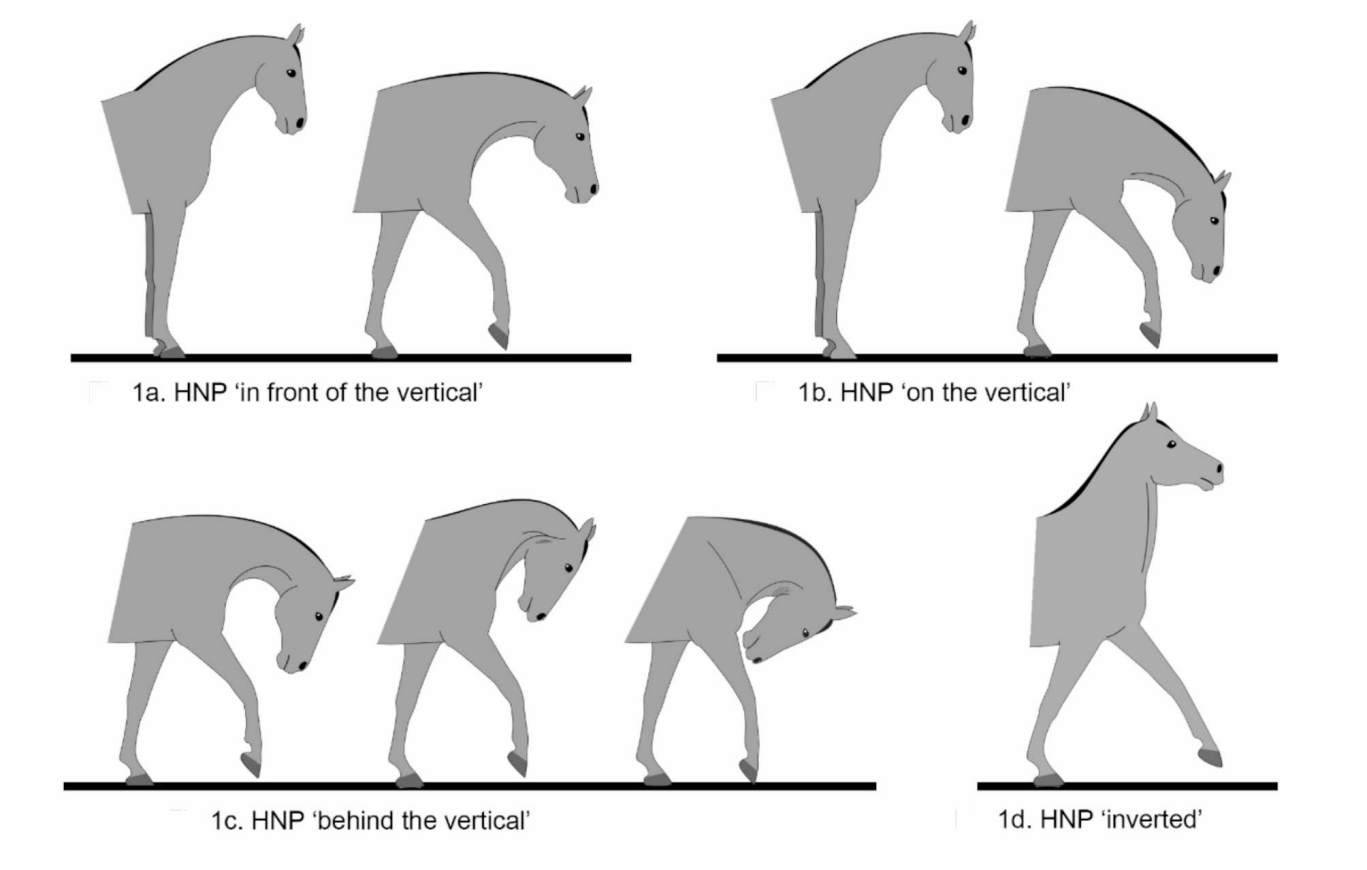
Different head–neck postures of horses, illustrated by Cristina Wilkins; courtesy International Society for Equitation Science (ISES).

Horses possess long, mobile necks that evolved to facilitate exploratory, social, and ingestive behaviours. Additionally, the neck plays a crucial role in locomotory behaviour, contributing to balance and coordination. In many equestrian activities, head and neck postures arising from positioning of the atlanto-occipital joint relative to the cervical vertebrae are valued and evoked by riders, trainers and coaches, typically via bit pressure (as a product of rein tension). The horse’s neck is often extremely flexed (see Fig. 1c) in a wide range of equestrian pursuits, including cross-country, dressage, show-jumping, driving and reining. Generally, these postures cannot be self-maintained by the horse and are seen in free-ranging horses for only brief periods, for example, to obtain relief from an itch. Following the rise of horse sports after the Second World War, sustained flexion with a nose-line behind the vertical has become increasingly common in equestrian contexts^1^. In the last few decades, techniques that involve extreme flexion of the neck for various periods have emerged as part of training, in particular, before a competition performance. It should be noted that in more recent years, some sport horse trainers have opted to forgo or limit the use of head-neck positions with the nasal plane behind the vertical. This shift coincides with a tougher stance from some dressage judges, who sometimes impose more severe penalties on horses ridden with their nasal plane behind the vertical in competitions. Additionally, stewards in warm-up arenas have, to some extent, followed suit. Nonetheless, extreme flexions of the neck are still commonplace, and the concern that head-neck postures, such as those illustrated in Fig. 1c, compromise horse welfare prevails. Unsurprisingly, the training practice is among several that compromise the profile of affected sports^2,3^.

Hyperflexion (or rollkur) is the term used to describe the training technique in which the horse’s neck is flexed dorsoventrally to various degrees, positioning the nasal plane or cranio-facial profile behind the vertical. In extreme cases, the horse’s chin may touch its pectoral region (see Fig. 1c). This practice has been associated with improved “submission” in horses and, as such, may give riders more control over highly reactive horses^4^. At this point, it should be emphasised that submission is a very poorly understood construct in horse training, as evidenced by the variability of scores it historically has attracted in elite dressage^5^. Moreover, there is the possibility that increased control over highly reactive horses may result from effectively diminishing their vision^1^. Visual diminution for putative behavioural outcomes is also seen in the use of blinkers in horse racing.

While hyperflexion is used in training and pre- and post-competition, the official judging guidelines stipulate that it should attract penalties when it is observed during a dressage competition, as the horse would be judged to be over-bent. The paradox of horses being warmed up in a position that judges are expected to penalise has been a topic of discussion in commentaries on the increased prevalence of elite horses performing behind the vertical (for example^6^). Studies have shown that this rule is not consistently applied in elite classes^7^.

Hyperflexion decreases stride length and increases elevation of the hindlimbs while also increasing the dorsoventral oscillation of the lumbar vertebrae^8^. Certainly, the current prevalence of hyperflexion among elite dressage competitors strongly suggests that it lends some competitive advantage in that its use helps to give a performance the judges reward, even though it runs counter to what is required in the competition itself.

The 2006 Fédération Équestre Internationale (FEI) workshop on hyperflexion drew attention to the effect of HNP on the respiratory tract of healthy horses and on vision, as well as other issues that surround the use of horses in modern dressage, including the general consequences of equestrianism on horse welfare^3,9^. The post-meeting report concluded there appeared to be “little scientific evidence that such practices actually result in lasting damage to the horse”. However, and despite the lack of available evidence, it states that “in sound, experienced professional hands at a top-level event, rollkur or rollkur-like practices are unlikely to cause lasting harm to a horse but may well cause discomfort and apprehension, and could therefore be a welfare concern”^3^.

The FEI appointed a veterinary committee and a welfare subcommittee to consult with stakeholders on this issue to identify what research was required to confirm the welfare impacts. In 2008, the welfare subcommittee subsequently withdrew its support for the practice, stating that hyperflexion in any equestrian sport was an example of mental abuse^10^. In response, the FEI has attempted to regulate, rather than ban, the practice, shifting the focus from the HNP itself to how it is achieved. Thus, in 2010, the FEI redefined hyperflexion or rollkur as flexion of the horse’s neck achieved through aggressive force^11^. This decision reflects an abiding lack of consensus over the use of terminology that contributes to ongoing confusion among riders, stewards and equitation scientists. The current ‘FEI Stewards Manuals for Jumping and Eventing’ include illustrations of permitted stretching techniques termed “low deep and round” (LDR), “long deep and round” (LDR) and “long and low” (LL)^12,13^. Additionally, the use of extreme hyperflexion (now referred to as ‘deliberate extreme flexions of the neck involving either high, low or lateral head carriages’) is permitted, providing it is performed for very short periods and is achieved by unforced and nonaggressive means. Stewards have been instructed to intervene, if extreme flexion is prolonged (i.e., exceeding approximately ten minutes^14^) or achieved through forced or aggressive riding, which are described in the rules as rough or abrupt cues or constant unyielding pressure on the horse’s mouth; as well as if the horse is in a state of general stress and/or fatigue. The current ‘Stewards Manuals for Dressage and Para-Dressage’ contain no reference to neck flexion in the forms described in the Jumping and Eventing Manuals; instead, they refer to ‘stretching the head and neck’ and ‘head/neck postures’, stipulating that they must be achieved as sensitively as possible and that continuous variation of head/neck posture is essential^15^.

Stewards in the warm-up arena are expected to make assessments of rein tension or a horse’s state of stress and/or fatigue without empirical data. This is problematic, given that trained judges have difficulty detecting rein tension as an attribute called lightness^16^, and equestrian professionals cannot concur on manifestations of stress in the ridden horse^17^.

Taken together, it becomes clear that the topic of hyperflexed head-neck postures is highly controversial in both science and practice, and the evidence regarding both, the welfare and the performance effects of training horses in this way is inconclusive. Proponents and opponents each cite studies that align with their views, while a comprehensive and conclusive assessment of the facts is lacking. The current article summarises the peer-reviewed studies that have examined the welfare, performance and physiological consequences of hyperflexion. It focuses on induced hyperflexion to distinguish anthropogenic practice from spontaneous flexion of the neck, as observed in free-ranging horses and those at liberty. In this article, we refer to head-neck postures as “HNP”. The cranio-facial profile or nasal plane is referred to as the “nose-line”. We hypothesised that the greater the degrees by which the horse’s nose-line is positioned behind the vertical (BV), and the longer they maintain this position (duration over 10 min), the more significant the negative welfare impacts and compromised performance-related effects they would experience, particularly affecting their upper airway and musculoskeletal function. Additionally, we proposed that higher-level dressage horses or those regularly trained in BV positions would exhibit less pronounced welfare issues when tested in comparison with horses unaccustomed to this practice.

## Materials and methods

### Review methodology

The review was conducted following the PRISMA guidelines and flow diagram^18^; i.e., the relevant literature was systematically identified, and the results were screened and assessed for eligibility. We used the existing peer-review system as the sole eligibility criterion apart from content-related considerations, because study design varies widely and it is impossible to implement the highest quality standards with regard to study design within applied research. For example, double-blinding is not possible for behavioural analysis in treatments that differ visually, as is the case with head-neck posture. We deemed any study eligible for inclusion if it was published in a peer-reviewed, scientific journal, reported original research, and addressed any aspect of HNP in horses. To account for publication bias, we also screened non-peer reviewed studies detected by the search (i.e. studies only published in conference proceedings or as part of a thesis). The literature search was independently conducted by two researchers (KK and UK) in the initial search phase, with each researcher conducting the search in half of the databases. The following databases were used (last search on 28.05.2023): CAB, Google Scholar, Web of Science, NAL/Agricola, PubMed, and ScienceDirect. In cases of uncertainty, both researchers collaboratively determined whether a given study was eligible for inclusion. In addition to these databases, the reference lists from the articles selected through the above process, and all the authors’ literature databases were used. The following keywords were used in various combinations for the search: horse, equine, riding, head neck position, head neck posture, poll flexion, hyperflexion, rollkur, and low-deep-and-round.

### Data preparation and processing

If an article included more than one experiment, each experiment was considered a separate study for the analysis so as to account for potential differences in experimental design. Based on the location of the experiment or the first/last author’s affiliation, each study was assigned to a laboratory. The identified articles were scanned for various characteristics regarding the type of article, experimental design, qualities of the horses and results.

The type of study was classified according to four categories: experimental (HNPs were induced experimentally), epidemiological (HNPs were observed as the main parameter and quantified in a sample of horses without being experimentally induced), byproduct (HNPs were not experimentally induced and not the focus of the study, but different HNPs were observed and related to other parameters assessed in the study), and survey (the study did not involve living horses, but human participants were surveyed on aspects including horses’ HNPs). From studies of the two categories “byproduct” and “epidemiological”, the prevalence of hyperflexed HNPs, if available, was noted to obtain an overview of different populations of horses.

For further analysis, binary data (i.e., aspect was investigated in the study or not) were created for the variables “high HNP”, “free HNP”, “halt”, “walk”, “trot and/or canter”, as well as for the different types of outcome variables investigated (such as behaviour, kinematics and airway functioning). Additional information collected regarding the study design included: the total number of horses studied; the number of horses in BV; the number of HNPs studied; the specific situation (for example, standing, riding, lunging, treadmill, picture); the method used to achieve HNP BV (for example, luring with feed, manual pressure on the horse’s nose, bit pressure via regular reins, bit pressure via side or draw reins, and various combinations of draw-reins, side-reins, and manual rein pressure, such as ridden with one type of rein and unridden with another); and the terms predominantly used by authors to refer to BV (for example, hyperflexion, rollkur, LDR, over flexion, extreme flexion, behind the vertical, flexion of the head and/or neck without a specific term, poll flexion, or only referring to angles). The duration for which BV was studied was converted into seconds and assessed as follows: for studies that used instantaneous measures (i.e., photographs), 1 s was assumed; for studies that stated a range of durations, the mean duration was assumed; for studies that stated a distance covered by the horse, approximate durations were assumed based on the gaits studied (halt, walk, trot and canter) with 1 gait cycle per second for the walk and 0.7s as mean duration for the trot and canter^19–21^, as these gaits were evaluated together as one category; and for field studies with data on competition rides and no duration given, 600 s (10 min) of test were assumed. The duration was consequently reported as an absolute, continuous value in seconds.

The type of hyperflexion investigated in a study was assigned to five categories: BV (deviation from the competition frame only by the nose-line being behind rather than at the vertical, i.e., the neck is elevated); LBV (nose-line behind the vertical with a lowered neck); BV + LBV (both of the above postures were studied separately in that study); AV (no hyperflexion but only poll flexion up to a posture with the nose-line at the vertical was studied); and IFV (nose-line in front of the vertical). The degree of BV aimed at in each study was noted in 4 categories: 0 = no BV investigated (see Fig. 1a + 1b), 1 = generally only mild deviations from the vertical (for example, if any deviation from the vertical was counted towards BV, see Fig. 1c, left and centre), 2 = extreme flexion was targeted but moderated, if horses were not compliant (aimed for, but not necessarily achieved, the degree of flexion shown in Fig. 1c right, regardless of head hight), 3 = flexion was induced to be as extreme as possible (postures measured to be equal to Fig. 1c right regardless of head hight) .

To facilitate statistical analysis, horses were assigned to four groups of approximately the same size according to their breed type: “Royal Dutch Sport Horse (KWPN)”; “warmbloods of various breeds (wb)”; “mixed breeds of horses including non-warmblood breeds (mix)” and “breeds for racing purposes”, i.e., Standardbreds and Thoroughbreds (racing)”. The horses’ level of experience in dressage was assigned to four categories: 0 [unridden] = horses had never been trained in dressage (for example, foals, race horses); 1 [non-dressage] = (the majority of) horses in the study had some basic dressage training but were not used for dressage competitions at an advanced level or higher (for example, show-jumping horses, leisure horses); 2 [competitive] = horses were competing at advanced national levels in dressage; and 3 [elite] = horses were elite dressage horses competing at an international level. In addition, information was collected on whether horses were accustomed to being ridden BV prior to the experiment or not (yes/no/unknown).

Based on each study’s results as well as their authors’ statements, each study’s findings were summarised to reflect their conclusions on the effects on (a) welfare and (b) performance. Welfare-related variables included behavioural and physiological stress parameters (for example, conflict behaviour, heart rate variability parameters, cortisol concentrations, eye temperature) and horse-rider interaction (rein tension, intensity of riders’ cues). Performance-related variables included measures of kinematics, musculoskeletal function, workload (when an overall higher workload was considered to be a desirable outcome in the interest of musculoskeletal conditioning), respiratory function and performance marks. Heart rate and airway impairment with evidence of air hunger were parameters that were considered to be relevant for both, welfare and performance. Results for welfare and performance were summarized as follows: [.] = no results regarding welfare or performance / aspect not studied; 0 = results insignificant or inconclusive; 1 = results indicate that training in BV has beneficial consequences on welfare or performance ; -1 = results indicate that training in BV has detrimental consequences to performance or welfare respectively. If the results were not fully conclusive but pointed in one direction, 0.5/-0.5 was assigned. Thereby, the various study designs, outcome variables and statistical methods could be subsumed to a common denominator. Some authors concluded effects on welfare or performance that were not directly supported by their data (for example, extrapolating to stronger degrees of BV or to other groups of horses and/or basing their arguments on data from previous studies). Therefore, in the current analysis we noted whether the conclusions were directly backed by the data reported within that study itself, which we termed as “study’s own data” (yes/no). Additionally, we noted whether the authors had suggested that welfare effects may be effective only if certain preconditions were met (for example, effects proposed to be applicable to unhealthy horses only). The current analysis also recorded whether the data or the authors suggested that the effects of head flexion were dose-dependent (i.e., the greater the degree of flexion the stronger the effects). Finally, the parameters studied were noted (rein tension, behaviour, performance, kinematics, stress physiology, heart rate/heart rate variability, workload, anatomy, and respiratory function); the parameters influenced by HNP were briefly described; and whether welfare effects, if any, were of a psychological or physiological nature or both.

### Interrater reliability

We tested the validity of our assessment by randomly selecting one-third of the studies based on a random number generator, then selected the study IDs for reassessment by a third rater and interrater-reliability calculation. Agreement between the original data table created jointly by two researchers (UKVB and KK) and the table created by an additional rater (CW) was 81.2% (mean agreement per variable ± 13.0% (SD)) of the results, demonstrating good agreement. Additionally, analysis using the core models for welfare and performance as well as the table including CW’s data were repeated, and no deviation of the results from the analysis of the original table was found; i.e., all insignificant effects remained insignificant.

#### Statistical analysis

Since the studies examined different outcome variables, comparing them directly wasn’t feasible because the effect sizes of these variables aren’t comparable. To address this, we assessed for statistical heterogeneity a subset of *n* = 10 studies reporting the most common outcome variable - heart rate. We compared heart rate in relation to HNPs BV versus control HNPs. Heterogeneity was measured using the I² statistic, as outlined by Higgins et al. (2003)^22^, and Hedge’s g was used to account for small sample sizes. Using the software SAS 9.4, we employed a generalised linear mixed model, assuming an underlying normal distribution to assess how various aspects of the study design and horses’ characteristics (i.e., all variables listed in Table 1 appendix) influenced each set of results in terms of welfare and performance. An investigation of the residuals revealed an insignificant deviation from a normal distribution (Kolmogorov‒Smirnov: *P* > 0.15) for both, welfare and performance, which verified our normal distribution assumptions were correct. The core model included the following fixed effects related to our main hypotheses: duration of BV, horses’ prior experience with BV, degree of BV, and horses’ dressage level. Laboratory-of-origin was considered as a random factor to account for experiments from the same authors or the same facilities having similar research questions and experimental designs. For the core model, the influence of duration of hyperflexion was tested as a categorical factor with two levels: less than 600 s and more than 600 s. These categories were established because 600 s was considered by the International Equestrian Federation to be an approximate limit for training dressage horses in a hyperflexed frame^14^. The remaining variables were introduced individually into the model either as covariables (duration, total number of horses, and number of horses in hyperflexion) or as fixed effects. To account for the risks of multiple testing (*n* = 30 variables), a Bonferroni correction was applied to the variables not considered in the core model to produce an adjusted significance level of *P* = 0.0017. In addition, two-way interactions between the degree, type, duration of hyperflexion, situation, way in which BV was achieved, dressage level and familiarity with BV were tested. The relative proportions and deviations of the distributions of study outcomes reporting implications for welfare and performance from uniform distributions were assessed with binomial tests and Chi-square statistics.

#### Ethical approval and adherence to guidelines

The present study did not involve testing of live animals. Therefore, no ethical approval was required for this study. For conducting the literature review, we followed the PRISMA guidelines^18^.

## Results

### Prisma

Through database searching, 82 records were identified, and an additional 13 records were identified through other sources. After removal of duplicates (*n* = 27), 68 papers remained. Another five papers were excluded during the screening process owing to their reporting of non-original research, leading to 63 full-text papers being assessed for eligibility, of which another seven full texts were excluded with reasons (for example, not providing any data on HNP effects). A total of 54 papers were included in both the qualitative synthesis and the quantitative synthesis (meta-analysis). Four of the identified papers reported on more than one experiment, all of which were counted as separate studies in the meta-analysis, resulting in a total of 58 studies (see appendix, Table 1). Eleven of these studies did not investigate an HNP BV and were thus included only in analyses regarding the gradual effect of neck and/or poll flexion. Thus, the final analyses encompassed 34 studies examining performance effects and 36 studies assessing welfare effects related to training BV. A total of eight studies not published in peer-reviewed, scientific journals were identified, of which three assessed welfare-related parameters in relation to BV, and which were used in assessment of publication bias. All three unpublished studies concluded negative welfare effects of training horse BV. The level of statistical heterogeneity was considerable (I²= 96% for the subset of studies reporting heart rate in relation to HNP BV versus control).

### Prevalence of horses ridden behind the vertical

All the studies showed that riding the horse with an HNP where the nose-line is behind the vertical is common practice, and the proportion of horses presented BV was approximately 2/3 of the horses in a dressage context; these findings are remarkably consistent across different horse populations (Table 1). The few exceptions to this high proportion of horses ridden BV arose in young, three-to-four-year-old stallions trained for performance testing (22%^23^), in dressage horses when viewed on a per-movement basis (34%^24^), in general riding horses presented in sales catalogues at a walk (40%) but not at trot or canter^23^ or at the Olympics three decades ago (45%) but not at the more recent Olympics (71%)^1,26^. In disciplines other than dressage, reports of horses that can be clearly assigned to another category (for example, jumping or eventing) are rare. Indeed, only two studies assessed the prevalence of BV in jumping horses or in a mixture of young dressage and jumping horses. The results of these studies differ markedly from those of other competitive dressage studies in terms of the proportion of BV (less than 10% ^27^ and 22%^23^, respectively).

**Table 1:** Overview and outcomes of the studies reporting prevalence of head-neck postures with the nose-line behind the vertical.

Source	Situation	% horses with nose-line behind the vertical
Kienapfel et al., 2014	Dressage competition, various national levels	69%
Kienapfel et al., 2022	Dressage competitions, international level	96.8%
McGreevy et al., 2010	Horses in sales catalogues	Overall 69% - Walk: 40% - Trot: 71% - Canter: 58%
Dyson et al., 2021	Elite dressage horses	66.7%
Bornmann et al., 2018	Sport horses presented in sales catalogues	58%
Lashley et al., 2014	Horses participating in Olympic dressage competition	Year 1992: 45% Year 2008: 71%
Hamilton et al., 2022	Lower-level British dressage competitions	34% (of movements)
König von Borstel et al., 2011	Stallions (3–4 years old) trained for performance test	22%
Gorecka-Bruzda et al., 2015	Elite dressage competitions	~ 50% (mean percentage of test duration)
Gorecka-Bruzda et al., 2015	Elite jumping competitions	< 10% (mean percentage of test duration)

### Welfare implications

**Fig. 2:**
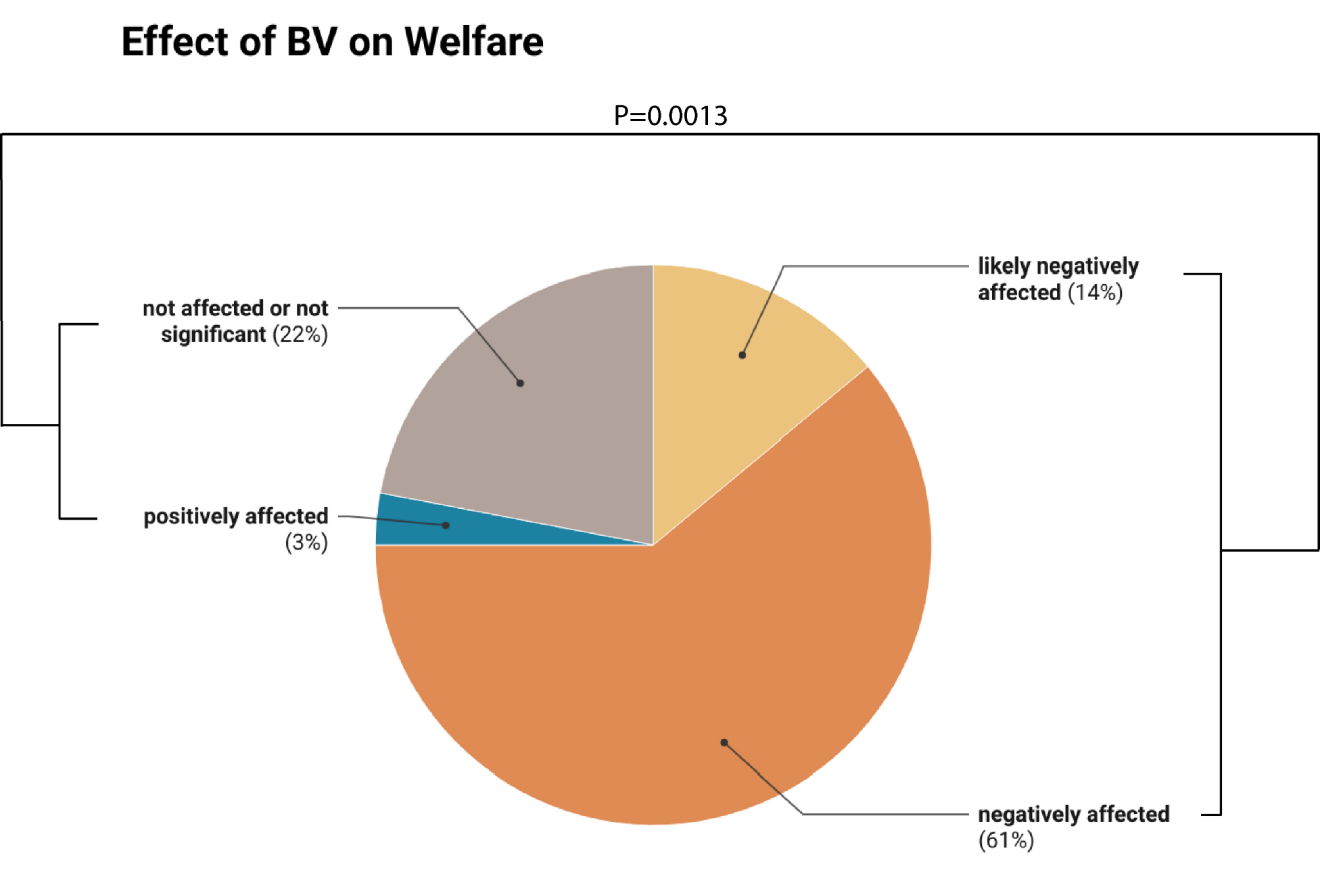
The proportion of studies discussing equine welfare in relation to head-neck postures with the nose-line behind the vertical (BV, *N* = 36) and concluding that horse welfare is negatively affected, likely negatively affected (conclusions are not (fully) supported by study’s own data), not significantly affected, or positively affected by training in BV. The proportion of studies suggesting that welfare is negatively affected (negatively + likely negatively: 75%) is significantly greater than the proportion of studies that reach alternative conclusions (not affected + positively affected: 25%; Z = 3.0; *P* = 0.0013).

A significant majority of the studies (75% of *n* = 36; Z = 3.00; P>|Z| = 0.0027) concluded that there are welfare implications of training in BV and/or LBV (Fig. 2). When considering only studies in which conclusions regarding welfare implications were clearly supported by the study’s own data, a significant majority (66.7%, *n* = 28, Z = 2.16; *P* = 0.0154) concluded that training BV impairs welfare. One study^24^ suggested potential welfare benefits for horses trained in this position.

Of the studies with results regarding BV, 22 (46.9%) indicated that the effects of neck flexion may be dose-dependent, with stronger flexion having greater effects on welfare and/or performance.

**Table 2:** Results of the analysis of factors affecting the welfare implications of training a horse in a head and neck position (HNP) with the nose-line behind the vertical (BV); meta-analysis of 36 peer-reviewed studies assessing welfare effects.

Core model	F value, degrees of freedom	*P* value (Significance level: *P* < 0.05)
Horses’ prior experience with BV (experienced, unexperienced, unknown)	F_2,7_=1.54	0.2783
Duration of training BV (≤ 600 s, > 600 s)	F_1,7_=1.50	0.2600
Degree of BV (4 categories*)	F_2,7_=4.18	0.0640
Horses’ dressage level (3 categories**)	F_3,7_=2.63	0.1318

*Degree of BV: 0 = no BV investigated, 1 = generally only mild deviations from the vertical (for example, if any deviation from the vertical was counted towards BV), 2 = extreme flexion was targeted, but moderated, if horses were not compliant, 3 = flexion was induced to be as extreme as possible. **Horses dressage level: 0 [unridden] = horses had never been trained in dressage (for example foals, racehorses); 1 [non-dressage] = (the majority of) horses in the study had some basic dressage training but were not used for dressage competitions at advanced level or higher (for example show-jumping horses, leisure horses); 2 [competitive] = horses were competing at advanced national levels in dressage; and, 3 [elite] = horses were elite dressage horses competing at an international level.

**Fig. 3:**
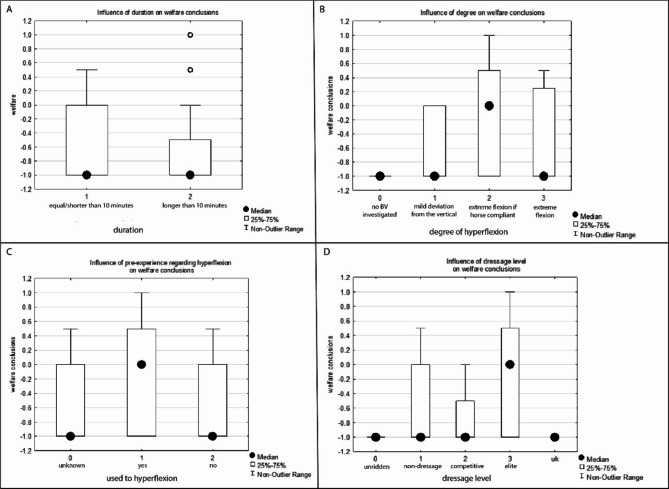
Relationships of (**A**) duration, (**B**) degree of hyperflexion, (**C**) prior experience with hyperflexion and (**D**) dressage level on welfare conclusion. None of the hypothesised factors significantly influenced the welfare implications of training the horse with the nose-line behind the vertical (all *P* > 0.1), i.e., training the horse behind the vertical deteriorates equine welfare regardless of the intensity to which it is requested or the horses’ characteristics.

No significant relationships were found between the hypothesised factors (duration, degree of head-neck flexion, dressage training level, or the horses’ pre-experiment exposure to hyperflexion) and the study’s conclusions on whether horse welfare is impaired during training in BV (all *P* > 0.1; Fig. 3).

Of the additional factors tested, none showed a significant (Bonferroni: *P* < 0.0017) influence on the studies’ conclusions about welfare. Therefore, negative welfare effects were observed regardless of the type of parameter used to study welfare effects (such as the type of study, the breed type, the way HNPs were generated or the gaits and situation used (standing, riding, lunging, treadmill, picture, other).

### Behaviour and horse-rider interaction

With the exception of one, 14 studies assessing behaviour in relation to HNP concluded that riding BV compromises horse welfare. The frequencies of reported conflict behaviours, such as tail-swishing and abnormal head and mouth movements, were significantly greater in horses ridden BV (for example^17,25–28^). In particular, oral behaviours correlated with HNP^7,28, 29^. Similarly, rein tension as well as the visually observable intensity of riders’ cues was greater during BV^25,26, 30–32^. When draw reins are used, their design may also amplify rein tension parameters^33^ and thus the intensity of riders’ cues. In addition, horses’ startle reactions tended to be stronger when ridden BV^25^, indicating that horses are in a state of higher anxiousness or hypervigilance when ridden BV, rather than AV. Similarly, less relaxation behaviour was documented in horses ridden BV rather than slightly in front of the vertical^28^. The prevalence of lameness was not associated with BV^34^.

### Physiological stress parameters

With a focus on physiological stress parameters, most studies revealed higher levels of stress in BV or no significant differences between BV and less flexed HNPs. For example, two studies detected higher concentrations of cortisol in horses ridden in BV^26,30^; one study found that cortisol concentrations correlated positively with low head carriage but not with head-neck angle^17^, and three studies did not detect any significant differences in cortisol concentrations^31,35, 36^. Similarly, in three studies, heart rate was higher during BV^25,28, 35^, or there were no significant differences in heart rate between BV and other HNPs despite the horses moving at lower speeds during BV^25^. Likewise, a study of HNPs other than BV did not detect any significant differences in heart rate at different HNPs^37^. Some studies detected changes in heart rate variability indicative of stress^31,38^, while other studies found no significant differences^26,36^. One study suggested that horses regularly trained in HNP BV had lower levels of stress during rest, as reflected by increased heart rate variability measured 30 min post-exercise^24^. Finally, in one study, there was a positive correlation between eye temperature and duration of BV, suggesting that horses perceived this posture as more stressful^17^. Overall, only one of the reviewed studies^24^ concluded that there may be welfare advantages of training BV compared to less flexed HNPs.

### Respiratory function and dysfunction

The effects of head flexion on upper airway structure and the potential for limits to breathing have clear implications for both welfare and performance. Airway impairment will be increased if there is narrowing of the airways due to structural airway collapse, and this will compromise welfare, for example, due to experiences such as air hunger^39^. However, since most studies assessing airway function focused on physical rather than psychological effects, the results regarding airway anatomy and function are discussed in detail below.

### Performance effects: Locomotion, musculoskeletal structure and function

**Fig. 4:**
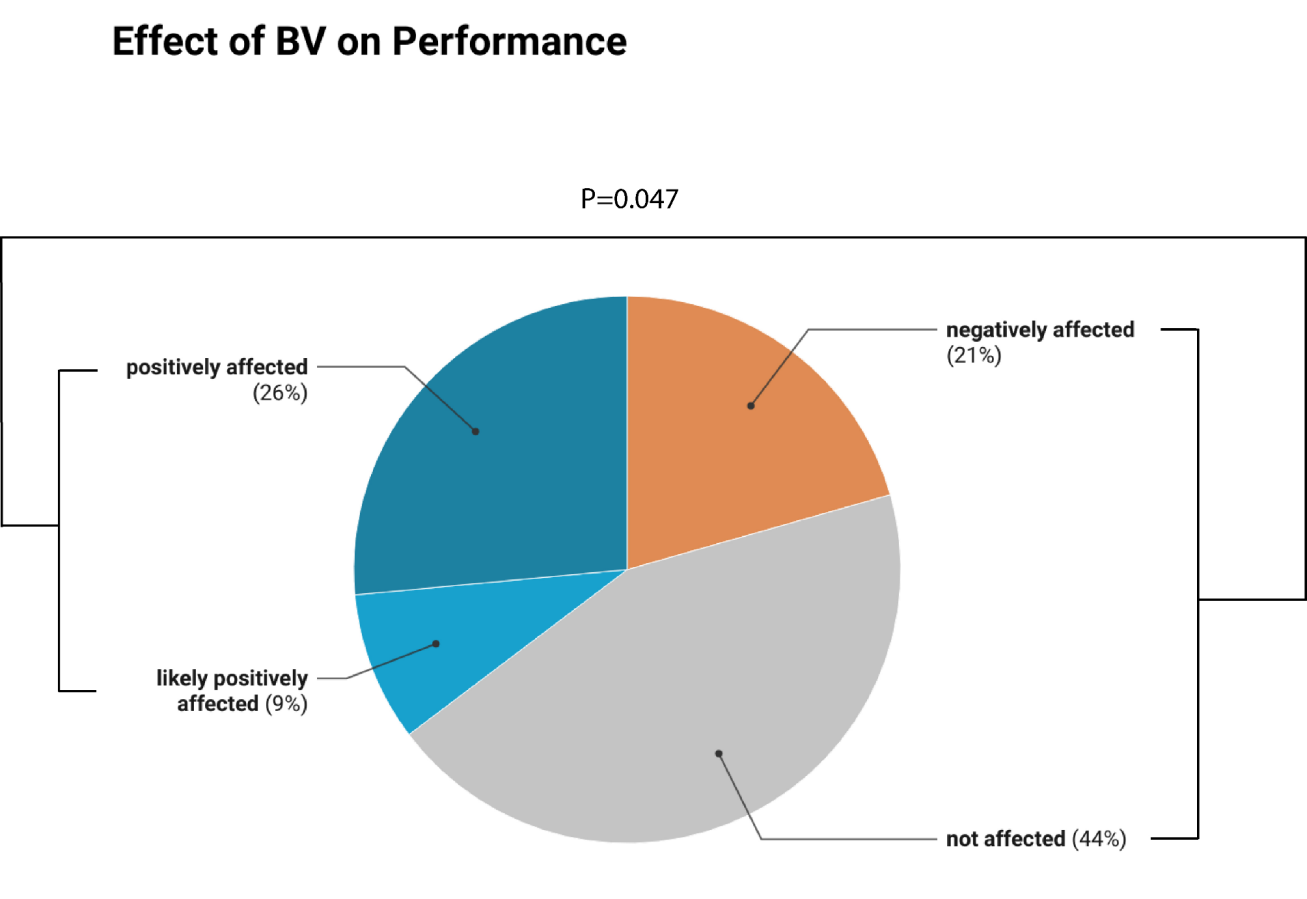
Proportion of studies investigating the effects of training horses with head-neck postures with the nose-line behind the vertical (BV) on performance (total *n* = 34), indicating that performance is positively (blue), likely positively (light blue), negatively (orange) or not (grey) affected. The distribution of proportions differs significantly from a uniform distribution (χ²=8.8; *P* = 0.0317). A significant majority (negatively affected + not affected; 65%) of the studies failed to find benefits for performance (Z = 1.7; *P* = 0.047).

Thirty-five studies investigated the effects of BV on performance. Compared with the welfare implications, the findings regarding the effects on performance are less clear (Fig. 4). The distribution of study conclusions significantly differed from a uniform distribution (χ²=8.8; *P* = 0.0317). However, the proportions of studies indicating performance benefits (*n* = 9) and those indicating undesirable effects (*n* = 7) of BV did not significantly differ from what would be expected by chance from a binomial distribution (Z = 0.5; Pr >|Z|=0.6171). In other words, there was no significant difference between the number of studies suggesting benefits and those suggesting undesirable effects. These outcomes did not change when including the three studies with inconclusive results in the group of studies suggesting performance benefits (*n* = 12 vs. 7 studies; Z = 1.15; Pr >|Z|= 0.2513). Additionally, a significant majority (65%) of the studies failed to find benefits for performance (Z = 1.7; *P* = 0.047). Reported performance benefits include higher performance marks when ridden BV^7,40–42^, reduced heart rate post-training compared to leisure horses^24^ and increased flexion-extension movement of some lumbar vertebrae^43^. Negative performance effects were identified as performance insufficiency due to decreased pharyngeal diameter^44^, excessive stretching of parts of the nuchal ligament^45^, high pressure at the sides of the nuchal bursa inducing a higher prevalence of nuchal bursitis^46^, high and irregular activity of the m. brachiocephalicus^47^, disturbance of blood flow in the neck as determined by thermography^36^, and an increase in incorrect postures (i.e. BV) in some equestrian disciplines^48^. Interestingly, a preference (93%) for nose-lines in front of the vertical was observed among participants in an online-survey.

**Table 3:** Results of the analysis of factors affecting performance when training a horse in a head and neck position with the nose-line behind the vertical (BV); meta-analysis of 36 peer-reviewed studies assessing performance effects.

Effect from core model	F value, degrees of freedom	*P* value (Significance level: *P* < 0.05)
Horses’ prior experience with BV (experienced, unexperienced, unknown)	F_2,8_=2.45	0.1481
Duration of training BV (≤ 600 s, > 600 s)	F_1,8_=1.99	0.1962
Degree of BV (4 categories*)	F_2,8_=0.11	0.8955
Horses’ dressage level (3 categories**)	F_3,8_=0.93	0.4703

*Degree of BV: 0 = no BV investigated; 1 = generally only mild deviations from the vertical (for example, if any deviation from the vertical was counted towards BV); 2 = extreme flexion was targeted but moderated if horses were not compliant; 3 = flexion was induced to be as extreme as possible; **Horses’ dressage level: 0 [unridden] = horses had never been trained in dressage (for example, foals, racehorses); 1 [non-dressage] = (the majority of) horses in the study had some basic dressage training but were not used for dressage competitions at advanced level or higher (for example, show-jumping horses, leisure horses); 2 [competitive] = horses were competing at advanced national levels in dressage; and 3 [elite] = horses were elite dressage horses competing at an international level.

As with the effects on welfare, none of the investigated factors significantly influenced the studies’ conclusions about performance effects associated with training the horse in BV (all *P* > 0.05 for factors of the core model and all P_Bonferroni_ > 0.0017 for remaining factors; Table 3).

### Judges’ marks in competition and correlation with horses’ behaviour and HNP

Five studies analysed the effect of HNP on scores in competitive riding, and with one exception^42^, these studies also evaluated the horses’ behaviour. Lashley et al. (2014) noted that, in recent times, the degree of head flexion correlated with the marks of elite dressage horses, such that the more riders rode their horses behind the vertical, the higher the awarded marks^42^. While some studies also observed these relationships at high^7,27^ and low^41^ national levels, these effects are not always present at the highest national dressage levels. Moreover, riders at low national levels scored lower in competition after warming up their horses BV^27^.

Mouth and tail behaviour had no influence on the judges’ scores^7,41^. In contrast, Dyson and Pollard^49^showed an overall correlation between conflict behaviour and BV with lower marks in high-level competitions, but as behavioural, kinematic and postural parameters were reported only as summarised variables rather than as individual parameters, further differentiation and comparison to other studies is not possible. In a survey that asked participants with and without an equine background about different neck shapes and HNPs, a natural head carriage with the nose-line in front of the vertical (for example Fig. 1a left) was favoured by participants^50^.

### Respiratory anatomy, function and dysfunction

The effects of training BV on aspects of respiratory function were investigated in 13 studies in the current review. Cehak et al.^44^ reported significant effects of HNP on the pharyngeal diameter. The largest pharyngeal diameter was found at the extended midway position, and the smallest pharyngeal diameter was found at the “dorsal, flexed position” (equivalent to Fig. 1b left). It was concluded that a decrease in the pharyngeal diameter limits airflow through the upper respiratory tract and may result in turbulence with subsequent dynamic airway collapse. The effect of HNP on the pharyngeal diameter during exercise was confirmed by Go et al., 2014^51^. The pharyngeal diameter was reduced in a BV position implemented by the rider in comparison to the unrestrained HNP^51^. However, an association between the pharyngeal diameter and the degree of head and neck flexion (described by ground and withers angle) was not confirmed^51^.

A study in resting horses used radiographic measurements and ground and wither angles to investigate whether the pharyngeal diameter differed among extended, neutral and “the established flexed position”^52^. The pharyngeal diameter, determined from lateral radiographs of 35 horses, differed significantly between the flexed (28.5 ± 9.6; all mean ± SD in mm), neutral (51.3 ± 8.87) and extended head and neck positions (55.6 ± 8.9).

Dynamic bilateral laryngeal collapse (DLC) was also associated with poll flexion in racing Standardbred horses^53^. Poll flexion altered the rostrocaudal positioning of the larynx and shrank the laryngeal lumen in harness racehorses affected with DLC. Poll flexion induced greater rostral advancement of the larynx in relation to the hyoid apparatus in resting Standardbreds affected by DLC, compared with controls. At the level of the vocal folds, poll flexion decreased the laryngeal lumen in horses affected with DLC compared with controls. In a study with 40 horses ridden just a few degrees BV, abnormalities of the upper airways were observed more frequently than in horses ridden with the nose-line in front of the vertical^28^. In addition, the intrathoracic pressure was greater and the pharyngeal diameter was smaller in horses ridden BV. However, in warmblood horses (*n* = 58), there was no significant association between the grade of laryngeal function during exercise and different HNPs^52^. Petsche et al. (1995)^54^ investigated the effect of HNPs on upper airway flow and mechanics in 5 horses. In the flexed position, inspiratory impedance increased, and inspiratory flow decreased at standardised reference points in the respiratory cycle. The authors concluded “that during strenuous exercise, head and neck extension has little effect on upper airway flow mechanics, but head and neck flexion causes upper airway obstruction to airflow”^54^.

### Physiological and kinematic influences

In unmounted, high-level dressage horses, stride length was reduced during walking in the hyperflexed and other positions compared with the unrestrained position, but there were no effects at the trot^55^. At a walk and both with and without a rider, asymmetry of vertical movement of the withers in dressage horses, was not different in hyperflexion compared with a natural position^56^. Comparatively high levels of nuchal ligament stretch were found in hyperflexion^45,57^ as well as a decrease in lamellar width^45^ and/or changes in tissue stretch and intervertebral angulations^58,59^. One study reported profoundly altered (compared with all other examined HNPs) electromyographic (EMG) activity pattern in the brachiocephalic muscle during hyperflexion in all gaits in BV, compared with competition and neutral frames, leading to more exaggerated front leg movements in that position^47^. In a cadaver study hyperflexion was shown to be associated with the highest pressures on the side of the nuchal bursa over the atlas and the longest nuchal ligaments compared with HNP in front of the vertical^46^.

## Discussion

### Prevalence of BV

In most of the studies reporting the prevalence of BV in practical application, approximately two-thirds of the horses are ridden with their nose-line behind the vertical. In the context of national (for example, the German Equestrian Federation (FN), Equestrian Australia (EA), and the Royal Spanish Equestrian Federation (RFHE)) and international (FEI) regulations, a remarkable inconsistency emerges. The national rules explicitly and repeatedly stipulate that the nose-line should be carried at or in front of the vertical line “at all times”. This was also the case for the FEI regulations until January 2023, when the sections describing desired postures and movements were removed from the rulebook and published as separate sport guidelines^60,61^. This begs the question of why the prevalence of this particular HNP continues despite the regulations and guidelines. Although not clearly shown by the present meta-analysis, training BV apparently lends some advantages to riders, such as stronger control over the horse and more exaggerated movements that are rewarded with higher dressage scores, at least at the elite level^7,42^. One study even indicated a trend of more frequent application of BV in elite dressage competitions compared to 20 years ago, despite the controversy surrounding BV that flared up in 2006 and persists to this day^42^. The putative advantages of riding BV must surely outweigh potential disadvantages and dangers, such as diminished deceleration effects owing to the dual function of rein pressure in providing not only the operantly conditioned basis for deceleration but also poll and neck flexion.

### Welfare implications

In the 2006 Report of the FEI Veterinary and Dressage Committee’s Workshop entitled “The use of over bending (“Rollkur”) in FEI competitions”, the authors concluded that even in sound, experienced professional hands at a top-level event, hyperflexion may cause “discomfort and apprehension” in the horse and could therefore be a welfare concern^62^. According to our meta-analysis, a significant majority of studies investigating behavioural and/or physiological indicators of stress in relation to HNP indicate that horses find being trained BV to be stressful. Although several studies failed to detect significant differences in physiological stress parameters between HNPs, it should be noted that the absence of a statistically significant difference does not confirm the absence of any effect^63^. In addition, none of the studies provide conclusive evidence of any welfare benefit from training BV. Furthermore, all unpublished studies examining welfare concluded negative welfare effects of training BV, suggesting that publication bias in a way that a disproportionate number of studies demonstrating positive welfare effects remain unpublished, is not an issue with regard to welfare effects^64^. Therefore, the current meta-analysis demonstrated that riding BV jeopardises equine welfare.

Questions about which factors, such as training level, prior experience of hyperflexion, or rider skill, influence potential welfare implications have long been debated (for example^64^). However, the current meta-analysis shows that welfare implications exist regardless of these factors. Nevertheless, 46.9% of the studies with results regarding BV suggested that the effects of head-neck flexion may be dose-dependent, with more flexion having a greater effect on welfare and/or performance. This finding could not be confirmed with the meta-analysis. It seems likely that our categorisation for the meta-analysis of the degree of flexion up to only three levels was insufficiently detailed to reveal a tipping point in such relationships. Certainly, the description and categorisation of HNPs differed widely among different studies, and many studies did not provide detailed information about the HNPs they investigated. This may be partly because many of the studies were conducted under practical conditions such that HNPs could not always be standardised accurately. In particular, the degree of poll flexion (i.e., the gullet angle) is likely a main determinant of welfare outcomes, and it would be important to have this information reported in future studies on HNPs. This is because when the head and neck are in a high position, even with a small degree of BV, the degree of poll flexion is extreme, whereas the gullet angle may be much wider with a low head-neck posture despite a larger degree of BV (compare Fig. 1c (left) with 1c (middle)). Having said this, it is important to note that negative welfare implications were also observed in studies investigating low HNP BV. Therefore, BV per se compromises welfare, likely for example due to psychological effects such as those arising from reduced forward vision^23^ and air hunger, even if physical strain due to extreme flexion is less prominent in such HNPs.

### Behaviour and horse-rider interaction

The majority of studies analysing behavioural parameters report increased frequencies of conflict behaviours such as tail-swishing and abnormal head and mouth movements^7,17, 25–29^, as well as heightened rein tension^65^ and rider aid intensity^25,31, 38^ when horses are ridden BV. These outcomes, along with stronger startle reactions and decreased relaxation^25,28^, suggest that horses experience greater stress and anxiousness in this position.

At this point, it is important to note that most behavioural observations in the analysed studies were made without controlling for the noseband or specifying its tightness, and for field studies, it can be assumed that the vast majority of nosebands were too tight^66^. Tight nosebands restrict oral conflict behaviour, and if nosebands were removed or fastened loosely, according to the two-finger rule (ideally using the taper gauge of the International Society of Equitation Science), the frequency of conflict behaviours would have been even higher^67^. However, in field studies using volunteer riders or participants from elite sports, such adjustments to the equipment by the researchers are usually not possible or not permitted.

Unlike the competition frame and other positions, the hyperflexed position typically requires the use of draw reins in many ridden trials across various studies^27,35, 36, 43, 56, 68–72^. This suggests that achieving and maintaining this position is challenging and may necessitate a level of force that volunteer riders are often unable or unwilling to apply^43,47, 55, 56, 72, 73^. This is supported by several studies showing that the more the horse’s poll was flexed, the greater the mean rein tension^32,74^.

In addition to the increased rein tension, riders also require higher intensities of leg and spur cues when riding horses BV, resulting in additional conflict behaviour in the horse. Similar to the increased intensity of rein cues, these amplified acceleratory cues often reach levels that cause discomfort and pain, and even tissue damage, which are clearly unacceptable from an animal welfare perspective^25,28, 75^.

### Physiological stress parameters

Regarding physiological parameters, most studies showed higher levels of stress in BV or no significant differences between BV and less flexed HNPs. However, it should be noted that in these studies, HNP was confounded with speed^25^, and that speed of movement is one major determinant of heart rate in exercising horses. Since horses prefer to move at a slower speed during BV than during less flexed HNPs, these non-significant differences in heart rate despite differences in speed may indicate greater emotional arousal in BV. These results are partly confirmed by studies detecting changes in heart rate variability indicative of stress^31,38^, although other studies did not detect significant differences^26,36^. Heart rate variability parameters as indicators of stress in exercising horses, have not yet been fully validated^76^. One study showed that horses regularly trained in HNP BV had lower levels of stress, as reflected by increased heart rate variability data measured 30 min post-exercise^24^. However, it is not clear how heart rate variability assessed 30 min post-training reflects experiences during training. Furthermore, in that study, treatment was confounded with factors such as training level and housing, and the resulting data are likely to reflect differences in these parameters rather than head posture per se.

At walk, stride length was reduced in the BV position compared with the unrestrained position^55^ in unridden horses. A decrease in stride length during walk in hyperflexion may be related to associated changes in muscle activity in the neck^47^, but direct cause-and-effect relationships have not been demonstrated. The key to ensuring equine welfare is that horses are enabled to use their bodies in ways that do not lead to lasting damage to anatomical structures. In BV, studies have shown changes in nuchal ligament length, tissue stretch and intervertebral angulation^58,59^ and a decrease in lamellar width, which was not present in the AV position^45^. Additionally, one study reported increased and altered neck muscle activity only in hyperflexion^47^, possibly leading to more exaggerated foreleg movements in that position, an attribute targeted in competitions and perceived as a performance benefit. Furthermore, a pathological examinations of 60 horses revealed insertion desmopathies in the squama occipitalis of all dressage horses^77^, suggesting that these pathological changes are caused by hyperflexion of the neck. That said, due to the study design, it could not be proven that HNPs are the cause of these changes^77^. Another study reported the highest pressure on the side of the nuchal bursa above the atlas and the greatest nuchal ligament length in hyperflexion compared with a HNP in front of the vertical^46^. While using an animal for leisure activities, requesting movements or postures endangering its integrity is unacceptable from an ethical point of view and prohibited by many national and international federations^60,78^, as well as welfare legislations^79,80^. Based on the current evidence, training horses BV increases the risk of tissue damage, a negative welfare outcome that is difficult to justify in the use of animals for sport.

### Respiratory anatomy, function and dysfunction

The findings on the effects of a flexed HNP on the pharynx and larynx differ markedly. Some studies reported reduced diameters in a flexed position in comparison to positions in which the head was carried more freely^28,44, 51–54, 81^. However, two studies found no clear association between HNPs and either the pharyngeal^52^ or laryngeal^82^ diameter. Overall, it seems that pronounced poll and neck flexion reduces laryngeal, as well as pharyngeal diameter. Clearly, the extent to which this compromises the health and welfare of sport horses of different disciplines requires further study. In particular, breathlessness and air hunger are expected to be highly aversive to animals^81,^ as air hunger is indeed unpleasant and deleterious to humans.

### Performance benefits

As with the welfare effects, none of the hypothesised factors significantly influenced the 35 studies’ conclusions about performance effects, i.e., longer durations, stronger degrees of BV, or familiarity with BV. Additionally, horses trained to a higher dressage level did not benefit more (or had more impairment) from training BV than other horses did. This finding contrasts with an earlier meta-analysis^83^, which included non-peer-reviewed studies and found that familiarity with BV had clear effects on performance. Specifically, horses trained in BV exhibited more exaggerated movements, possibly as post-inhibitory rebound response to BV.

In conclusion, gait quality at the walk and breathing ability are influenced by HNP. Head and neck flexion cause changes in soft tissue tension and muscle activity in the neck. These findings raise concerns about the welfare of horses forced to exercise with their head and neck in hyperflexion.

### Judges’ marks in competition

It is imperative to recognise that judges are pivotal gatekeepers of horse welfare. Notably, the effect of HNPs on the marks that judges award in practice appears to depend on the dressage level. While riders at the lower national levels were marked down in dressage competitions when riding BV, those at the highest national or international level were either not marked down for riding their horses in this posture^27^ or were awarded higher marks when riding more BV^41^. Similarly, stallions ridden in BV for a larger proportion of time during their performance tests (here basic dressage training for young, but mostly highly talented horses) received higher scores for rideability and personality overall^40^. While these findings might provide, at first sight, support for the popular claim that only skilled riders are able to practice this riding style without detrimental effects on the horse, the observations by Zebisch et al. (2013)^30^ indicate that the higher the training level is, the more behaviours indicative of conflict horses exhibit during riding BV^30^ and that, regardless of skill, the prospect of air hunger remains. Kienapfel et al. (2014)^27^ also found higher frequencies of behaviour indicative of conflict in dressage horses at higher rather than lower training levels, with the highest frequencies of all being present at the elite level. Overall, these findings suggest that judges apply the rules inconsistently across different groups of horses and riders. Conflict behaviour and HNPs at odds with the official rules led to penalties less frequently for riders at higher levels. Interestingly, a study reported that the majority (93%) of survey participants favour HNPs with natural head carriage, which is clearly in contrast to judging preferences^50^. However, it is important to note that the findings of another study apply to more recent performances (i.e., 2008) but not to historical performances (i.e., 1992), indicating a shift in judges’ perceptions of HNPs^42^. It is also relevant that at higher levels of competition, judges are evaluating more complex and frequent movements, with a focus on horses’ feet^84^. Therefore, HNP may receive more attention at lower levels, where there are fewer movements to assess and the rider is more in focus^84^.

### General remarks and limitations

The current study drew on a large number of studies with markedly heterogeneous study designs, often adapted to accommodate practical considerations. So, it was difficult to reduce all these diverse studies to a common denominator that allowed complete across-study analysis. For example, there is no standardised protocol for defining HNPs across studies. Some studies, mainly those conducted under practical conditions with limited data collection possibilities, have only distinguished between HNP BV and one or more non-BV HNPs. In contrast, other studies have taken great care to precisely define both poll and neck angles. While such detailed definitions or recordings of HNPs are preferable, it should be noted that, in practice, these precise angles often cannot be maintained for an extended period. Instead, due not least to the movement of the horse, they vary in terms of degrees of flexion. Ideally, representative samples taken throughout any study should be used for assessment and later reporting of effectively achieved nose-line, poll and neck angles.

Another limitation of analysing HNPs emerges due to the lack of technical equipment for measuring HNPs in live setups, as well as for analysing HNPs later in video. The latter approach is especially common when markers are not used or cannot be used. In a field study design, the application of sensors is usually not feasible. In addition, reliable or affordable equipment for measuring HNPs is scarce. The advent of deep learning models for detecting emotional states in the ridden horse should allow the fresh analysis of video footage from competitions at various levels^85^.

Furthermore, for the present meta-analysis, applying high-level quality inclusion criteria, as required for Cochrane reviews was not feasible^86^. This is because nearly all available studies encountered issues such as small sample sizes (for example, with the exception of one study, none justified the chosen sample size), a lack of external validity owing to highly selected sample populations, and potential bias arising from the inability to employ double-blinded study designs. Also, publication bias may be present, such that studies with insignificant or unexpected outcomes are less likely to be published. It is difficult to quantify the risk of publication bias, but given the overwhelming evidence for negative welfare effects of hyperflexion in both the published studies and the set of unpublished studies, plus the fact that there is a large group of riders with a vested interest in continuing the practice of hyperflexion, it seems unlikely that an even larger number of studies with positive welfare findings remained unpublished. Moreover, the internal validity of some studies appears questionable when relying on parameters that are not well established as markers of stress within the given sampling protocol. Considering these challenges, excluding individual studies based on these criteria alone would involve highly subjective judgements due to the heterogeneity of the study designs, which is also reflected by considerable differences in effect sizes and as a result the very large I² value. Consequently, we opted to use the existing peer-review system as the sole criterion for quality inclusion.

Some studies^53,54, 87, 88^ did not analyse BV but only HNPs with the nose-line at the vertical. Their results on welfare and performance were excluded, but they nevertheless met the criteria for general inclusion in this meta-analysis. Four out of five of these studies concluded that there are undesirable effects (regarding either welfare or performance effects or both) of head and neck flexion alone, even without being BV. It was also concluded in one study that even moderate head flexion can lead to health and/or welfare problems, especially in horses with a history of respiratory disease^87^. This is an interesting finding and should be considered when discussing the influence of HNPs on horses.

## Conclusions

1. The consensus is that there are negative welfare consequences for horses required to perform with a hyperflexed HNP.
2. Seventy-five per cent of the peer-reviewed articles raised concerns about the welfare of horses working in this posture, and the current meta-analysis revealed that welfare implications arise irrespective of factors such as the horses’ level of dressage training, prior experience with the posture, the way hyperflexion was achieved, or the duration or degree of hyperflexion.
3. A concurrent assessment of the evidence for performance benefits from hyperflexing horses’ necks showed that a significant majority (65%) of studies failed to find benefits of training horses in this way.
4. On balance, it appears that the costs associated with training BV exceed the potential benefits of the activity for horses. This finding should inform cost‒benefit considerations and ethical decision-making around these techniques in equitation^89^.
5. When animals are used in sports, our ethical responsibilities are extraordinary, and these responsibilities coupled with the sustainability of such sports require ongoing reassessment of common practices^90^. The current meta-analysis is timely and should be reflected in the rules that govern horse sports in tandem with the education of judges.

## Electronic supplementary material

Below is the link to the electronic supplementary material.

Supplementary Material 1

## Data Availability

The compiled data table on which the meta-analysis is based, is available at the following link: doi.org/10.5281/zenodo.10820505.
